# CD127 Expression, Exhaustion Status and Antigen Specific Proliferation Predict Sustained Virologic Response to IFN in HCV/HIV Co-Infected Individuals

**DOI:** 10.1371/journal.pone.0101441

**Published:** 2014-07-09

**Authors:** Hassen Kared, Sahar Saeed, Marina B. Klein, Naglaa H. Shoukry

**Affiliations:** 1 Centre de Recherche du Centre Hospitalier de l'Université de Montréal (CRCHUM), Montréal, Quebec, Canada; 2 Department of Medicine, Divisions of Infectious Diseases/Chronic Viral Illness Service, Royal Victoria Hospital, McGill University Health Centre, Montreal, Quebec, Canada; 3 Département de médecine, Faculté de médecine, Université de Montréal, Montréal, Quebec, Canada; KAIST, Graduate School of Medical Science & Engineering, Republic Of Korea

## Abstract

Hepatitis C virus (HCV) infection is a major cause of morbidity and mortality in the HIV co-infected population. Interferon-alpha (IFN-α) remains a major component of anti-HCV therapy despite its deleterious effects on the immune system. Furthermore, IFN-α was recently shown to diminish the size of the latent HIV reservoir. The objectives of this study were to monitor the impact of IFN-α on T cell phenotype and proliferation of HIV and HCV-specific T cells during IFN therapy, and to identify immune markers that can predict the response to IFN in HICV/HIV co-infected patients. We performed longitudinal analyses of T cell numbers, phenotype and function in co-infected patients undergoing IFN-α therapy with different outcomes including IFN-α non-responders (NR) (n = 9) and patients who achieved sustained virologic response (SVR) (n = 19). We examined the expression of activation (CD38, HLA-DR), functional (CD127) and exhaustion markers (PD1, Tim-3, CD160 and CD244) on total CD4 and CD8 T cells before, during and after therapy. In addition, we examined the HIV- and HCV-specific proliferative responses against HIV-p24 and HCV-NS3 proteins. Frequencies of CD127^+^ CD4 T cells were higher in SVR than in NR patients at baseline. An increase in CD127 expression on CD8 T cells was observed after IFN-α therapy in all patients. In addition, CD8 T cells from NR patients expressed a higher exhaustion status at baseline. Finally, SVR patients exhibited higher proliferative response against both HIV and HCV antigens at baseline. Altogether, SVR correlated with higher expression of CD127, lower T cell exhaustion status and better HIV and HCV proliferative responses at baseline. Such factors might be used as non-invasive methods to predict the success of IFN–based therapies in co-infected individuals.

## Introduction

Approximately 25% of all human immunodeficiency virus (HIV) infected individuals are also co-infected with hepatitis C virus (HCV) [1], [2]. HIV infection accelerates the natural history of HCV and liver disease progression. Combination of anti-retroviral therapy (cART) has decreased mortality among HIV-infected individuals but rendered the effect of HCV-induced liver damage more visible and it is now a major cause of mortality in this population [3]. The risk of liver failure is estimated to be 6 fold higher in co-infected individuals as compared to HCV mono-infected individuals [4]. This accelerated natural history correlates with the decline in CD4 T cell counts. The reduced frequency of helper CD4 T cells during HIV infection contributes to a reduction in HCV-specific humoral [5] and cellular responses in co-infected patients [6], [7]. HCV/HIV co-infected patients exhibit higher circulating HCV RNA in peripheral blood [8]–[10], reduced rate of spontaneous resolution of HCV infection [11], [12] and lower responsiveness (up to 30%) to IFN-based therapy [13], [14].

Depletion of CD4 helper T cells was shown to correlate with loss of mucosal integrity and increased microbial translocation [15] and consequently immune activation induced by HIV infection [16]–[19]. The T cell activation levels observed during co-infection are greater than those observed in chronic HIV patients [20]–[22]. Microbial translocation observed in co-infected patients is also a negative predictor for an early virologic response to HCV therapy [23]. Taken together, these observations suggest an active influence of HCV viral replication in sustaining immune activation and reducing responses to anti-HCV therapy.

Despite the successful development of direct acting anti-virals (DAAs) for the treatment of HCV, IFN-α remains a major component of current treatment regimens. Recent reports have demonstrated that IFN-α has significantly reduced the size of the latent HIV reservoir and suggested that it could have a beneficial role in achieving complete HIV cure [24], [25]. Nevertheless, IFN-α has multiple side effects that can be deleterious for HIV infected individuals as it induces pan T cell lympho-cytopenia and has a profound effect on thymopoeisis. Although, CD4 lympho-cytopenia may complicate treatment course and induce anemia [26] it has not been associated with opportunistic infections [27]–[29]. In this study we examined the effect of IFN-α therapy on the maturation, activation and exhaustion status of CD4 and CD8 T cells, as well as HCV- and HIV-specific T cell responses. We demonstrate that the activation and exhaustion status of T cells were predictive of IFN-α responsiveness in HCV/HIV co-infection. The success of IFN-α-based therapy was associated with higher basal expression of CD127 and antigen-specific proliferation of HCV- and HIV-specific T cells.

## Patients and Methods

### Ethics statement, study subjects and clinical follow-up

The Canadian Co-infection Cohort Study (CCC) is a prospective open cohort of HCV/HIV-co-infected patients recruited from 16 centers across Canada [30]. This study is approved by the Biomedical B Research Ethics Board of the McGill University Health Centre (Protocol No. BMB-06-006t). Written informed consent was obtained from all participants. Eligible participants are adults aged 16 years and older (the legal age of informed consent in Quebec) with documented HIV infection (ELISA with western blot confirmation) and with chronic HCV infection or evidence of HCV exposure (e.g. HCV-seropositive by enzyme-linked Immunosorbent assay (ELISA) with recombinant immunoblot assay II (RIBA II) or enzyme immunoassay (EIA) confirmation, or if serologically false negative, HCV RNA-positive). The study was conducted on blood specimens from 28 individuals from the CCC who received HCV treatment between 2003 and 2010. The patients included 4 women, 23 men and one transgender patient. Patient demographics are listed in Table 1. Each patient received IFN-based treatment (IFN-α 2a or 2b + ribavirin) for a planned 48 weeks irrespective of HCV genotype. Patients who demonstrated <2 log decline in HCV viral load at week 12 or who had a positive HCV RNA at week 24 were considered treatment non-responders and stopped treatment. Patients who demonstrated ≥2 log decline in HCV viral load continued another 36 weeks of treatment if they became HCV RNA negative thereafter. The study was approved by the research ethics boards of the participating institutions. HLA typing was performed as previously described [31].

**Table 1:** Demographics and Clinical Characteristics of HCV-HIV co-infected patients at Recruitment^a^.

	Group A (HCV/HIV)^b^ (n = 14)		Group B (HIV/HCV)^c^ (n = 14)	
	NR (n = 4)	SVR (n = 10)	NR (n = 5)	SVR (n = 9)
Male gender	4 (100%)	9 (90%)	5 (100%)	5 (55%)^d^
Age (yrs)	45 (32–60)	46 (37–56)	45 (41–52)	36 (31–64)
Estimated duration of HIV infection (yrs)	8 (2–15)	9 (0.5–20)	15 (3,5–24)	16 (6–46)
CD4 count (cells/mm^3^)	376 (260–435)	320 (110–880)	460 (307–529)	407 (181–962)
Estimated duration of HCV infection (yrs)	24 (4–37.5)	22 (2–31)	8 (2–24)	6.5 (0.5–20)
HCV genotype (1/2b/3a/4/ND)	2/0/1/0/1	6/2/1/0/1	3/0/0/1/1	5/1/1/1/1
HCV viral load (IU/ml)	8×10^6^ (0.6–15×10^6^)	15×10^6^ (0.8–54×10^6^)	2×10^6^ (0.3–3×10^6^)	6×10^6^ (0.7–13×10^6^)
Serum ALT (U/L)	94 (68–262)	42 (28–353)	54 (43–95)	50 (6–612)
APRI score a	0.88 (0.7–2.14)	1.42 (0.15–4.07)	0.77 (0.22–1.29)	0.54 (0.32–3.48)

a Median and range values at recruitment.

b This group acquired HCV prior to HIV infection.

c This group acquired HIV prior to HCV infection.

d This group had one transgender patient.

### Flow cytometry antibodies and reagents

Directly conjugated antibodies against the following surface molecules were used: CD4-PerCP (clone SK3), CD8-APC-H7 (clone SK1), PD1-FITC or-PE (clone MIH4), CD244-FITC (clone 2-69), CD38-PE-Cy7 (clone HIT2) and HLA-DR-A700 (clone G46-6) (all from BD Biosciences, San Jose, CA); CD127-eFluor 450 (clone eBioRDR5), CD160-Alexa 647 (clone BY55) (both from eBioscience); CD3-ECD (clone UCHT1) (Beckman Coulter, Marseille, France); Tim-3-PE or –PerCP (clone 344823) (R&D Systems, Minneapolis, MN). Live cells were identified using Aqua Live/Dead Fixable Dead Cell Stain Kit according to the manufacturer's protocol (Life Technologies, Burlington, ON). “Fluorescence minus one” control stains were used to determine background levels of staining. Multi-parameter flow cytometry was performed using a standard BD LSR II instrument equipped with blue (488 nm), red (633 nm), and violet (405 nm) lasers (BD Biosciences) to systematically perform 9-11 color staining using FACS Diva software (Version 6.0.3) (BD Biosciences). Compensation was performed with single fluorochromes and BD CompBeads (BD Biosciences). Data files were analyzed using FlowJo software, version 9.4.11 for Mac (Tree Star, Inc., Ashland, OR).

### Phenotypic characterization of virus-specific T cells using MHC class I tetramers and CFSE proliferation

MHC class I tetramers were synthesized by either the National Immune Monitoring Laboratory (NIML) (Montréal, QC, Canada), the NIH Tetramer Core Facility (Emory University, Atlanta, GA, USA) or purchased from Proimmune (Pentamers, Oxford, UK) and Beckman Coulter (iTAg MHC tetramers, Mississauga, ONT, Canada). The following tetramers were used to analyze the CMV-, HCV- and HIV-specific CD8 T cell responses based on the patient's HLA: CINGVCWTV (A2/NS3-HCV), KLVALGINAV (A2/NS3-HCV), ALYDVVTKL (A2/NS5b-HCV), GPRLGVRAT (B7/core-HCV), NLVPMVATV (A2/pp65-CMV), TPRVTGGGAM (B7/pp65-CMV), SLYNTVATL (A2/p17-HIV), TLNAWVKVV (A2/p24-HIV), LTFGWCFKL (A2/Nef-HIV), SPRTLNAWV (B7/p24-HIV), TPQDLNTML (B7/p24-HIV), HPVHAGPIA (B7/p24-HIV) and TPGPGVRYPL (B7/Nef-HIV). All flow cytometry assays were performed on cryo-preserved samples. Phenotypic analysis using tetramers was performed as previously described [32]. CFSE proliferation assays were performed as previously described [33] for 6 days with or without 1 µg/ml HCV recombinant protein NS3 or HIV recombinant protein p24 (Feldan, Quebec, QC, Canada) in the presence of 200 ng/ml anti-CD28/-CD49d (Fastimmune, BD bioscience) at 37°C and 5% CO_2_. CMV and SEB stimulation were used as positive controls for proliferation of T cells. Recombinant human IL-2 (20 IU/ml) (NIH AIDS Research and Reference Reagent Program, Germantown, MD) was added on day 3. On day 6, cells were directly stained with surface antigens as described above.

### Statistical analysis

All analyses were performed using GraphPad Prism version 5.0 (GraphPad Software, San Diego, CA, USA). The Mann-Whitney rank sum test was performed to compare median values between two groups. Wilcoxon signed rank test was used to examine longitudinal statistical analysis. Correlations were determined using Pearson correlation test. P-values <0.05 were considered significant.

## Results

### IFN-α treatment induces reduction in CD8 T cell numbers and limits fibrosis in SVR patients

We examined the effect of IFN-α therapy on the immune functions of a group of 28 HCV/HIV co-infected patients recruited through the Canadian co-infection cohort study (CCC). Patients' characteristics and demographics are listed in Table 1. Within that group, 14 patients had acquired HCV prior to HIV infection, hereafter referred to as group A and 14 patients had acquired HIV prior to HCV, hereafter referred to as group B. All patients were on cART and initiated a 48 week pegylated-IFN-α 2a or 2b + ribavirin therapy according to the Canadian guidelines at the time as detailed in Materials and Methods. Patients who demonstrated <2 log decrease in viral load at week 12 or tested HCV RNA positive at week 24 discontinued treatment and were classified as IFN non responders (NRs). Patients testing HCV RNA negative 24 weeks after the end of treatment were classified as sustained virologic responders (SVRs). Relapser patients were not included in this study. Immune responses were analyzed in peripheral blood mononuclear cells (PBMCs) collected at three time points: baseline prior to initiation of therapy, 12 weeks post initiation of therapy and 24 weeks post termination of therapy whether it occurred at 48 weeks for SVR patients or earlier for NR patients.

First, we evaluated the overall change in T cell numbers in the different groups. Pre-treatment total lymphocytes, CD4 or CD8 T cell numbers were not different between the two groups. As expected with HIV infected individuals, we observed an inversed CD4/CD8 ratio. We observed a mild decrease in the CD4 numbers in SVR patients during therapy (p = 0.05). Similarly, CD8 numbers were significantly reduced in the SVR group during treatment (p = 0.01) and remained so after treatment (p = 0.05) (Figure S1). Moreover, liver fibrosis was measured indirectly by the aspartate amino-transferase to platelet ratio index (APRI) score [34]. APRI score was notably reduced in SVR patients after IFN-α therapy (p = 0.04) (Figure S2), but is likely due to systemic reduction in liver inflammation and reduction of serum AST as platelet numbers remained unchanged in most patients (data not shown)

### Sustained virologic response to IFN-α therapy correlates with baseline expression of CD127 and Tim-3 on CD4 T cells

**Figure 1:**
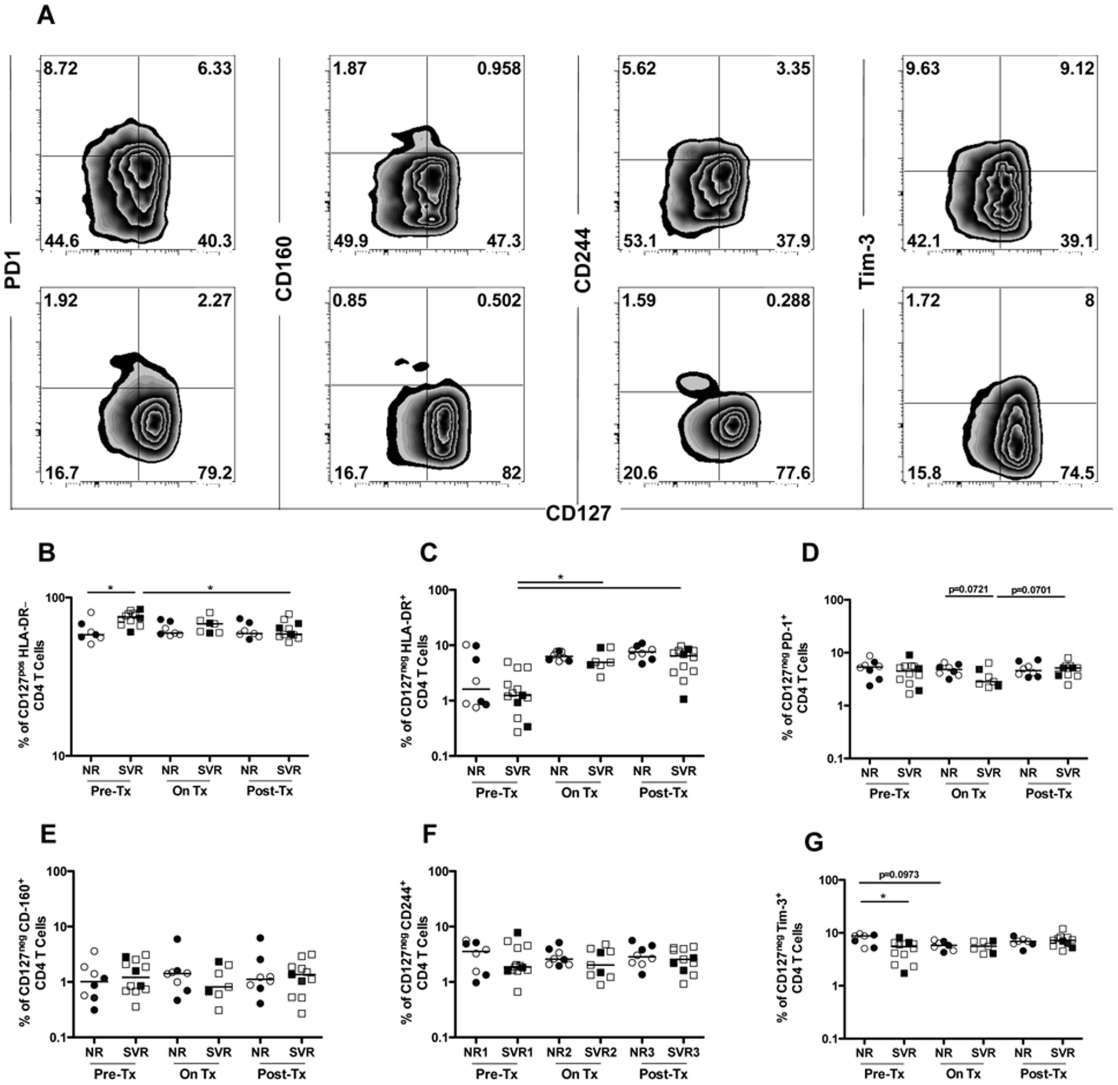
Sustained virologic response to IFN-α therapy correlates with baseline expression of CD127 and Tim-3 on CD4 T cells. Expression of the memory marker CD127 and the indicated activation or exhaustion molecules on CD4 T cells was monitored longitudinally in NR (n = 7) and SVR (n = 12) HCV/HIV co-infected patients before, during and after IFN-α therapy. (A) Representative flow cytometry plots for one patient from each group NR (top panel) and SVR (bottom panel) demonstrating expression of the different markers on total CD4 T cells (gated on CD8^neg^CD3^+^ lymphocytes). (B) Expression of the memory marker CD127; (C-G) CD4 activation/exhaustion was measured by the absence of CD127 and the expression of the activation/exhaustion markers HLA-DR, PD-1, CD160, CD244 and Tim-3. P-values were calculated using a two-tailed Mann Whitney U test to compare NR to SVR patients. Wilcoxon signed rank test was used to perform longitudinal statistical analysis. Open symbols represent patients of group A and closed symbols represent patients of group B. (* p<0.05).

Although we did not observe a major change in CD4 T cell numbers during and following IFN-α therapy, we reasoned that there might still be a change in the activation and differentiation status or distribution of the different CD4 T cell subsets. It is well established that HIV continues to replicate in activated CD4 T cells [35] and persists as a latent reservoir in resting CD4 T cells [36], which may influence their helper functions. We thus proceeded to examine expression of activation and exhaustion markers on CD4 T cells before, during and after IFN-α therapy. According to published literature [37], [38], we defined resting CD4 T cells as CD127^+^HLA-DR^neg^ and activated CD4 T cells as CD127^neg^HLA-DR^+^. The frequency of CD38+HLA-DR+ CD4 T cells was very low given that patients were all on cART and did not undergo significant changes during IFN treatment (data not shown). In addition, we examined the expression of the exhaustion markers PD-1, CD160, CD244 (2B4) and Tim-3 on activated CD4 T cells (Figure 1A). The frequency of CD127^+^HLA-DR^neg^ CD4 T cells was higher in SVR than in NR patients at baseline (p = 0.02) (Figure 1B). This frequency decreased slightly in SVR patients during therapy and was statistically significant post treatment (p = 0.02). The cause of this loss in CD127 expression could be explained by the activation of T cells due to IFN-α residual viral replication or microbial translocation [15]. Although, we observed no difference in the frequency of activated CD127^neg^HLA-DR^+^ CD4 T cells (Figure 1C) between NR and SVR patients at baseline, the frequency of activated cells increased in the SVR group during therapy (p = 0.05) and these activated cells persisted post treatment (p = 0.03). The analysis of the exhaustion markers PD1 (Figure 1D), CD160 (Figure 1E) and CD244 (Figure 1F) did not demonstrate any difference between the groups at baseline or over time. Tim-3 was the only exhaustion marker that was differentially expressed between NR and SVR patients at baseline, being higher in NR patients (p = 0.02) but this difference became insignificant during IFN-α therapy (Figure 1G). Hence, the activation and exhaustion status of CD4 T cells may correlate with the response to IFN-α therapy.

Next, we investigated the correlation between activation status of CD4 T cells and liver fibrosis before and after IFN-α therapy. We observed a negative correlation between frequencies of CD127^+^HLA-DR^neg^ CD4 T cells and APRI score before (p = 0.0033, r = -0.6523, n = 18) and after (p = 0.02, r = -0.5331, n = 18) IFN-α therapy (Figure S3A, C). Conversely, expression of the inhibitory receptor Tim-3 on CD4 T cells correlated positively with liver damage but only before treatment (p = 0.0083, r = 0.6011, n = 18) (Figure S3B, D).

### Sustained virologic response to IFN-α therapy correlates with higher baseline expression of CD127 while non-response correlates with increased CD8 T cell activation

**Figure 2:**
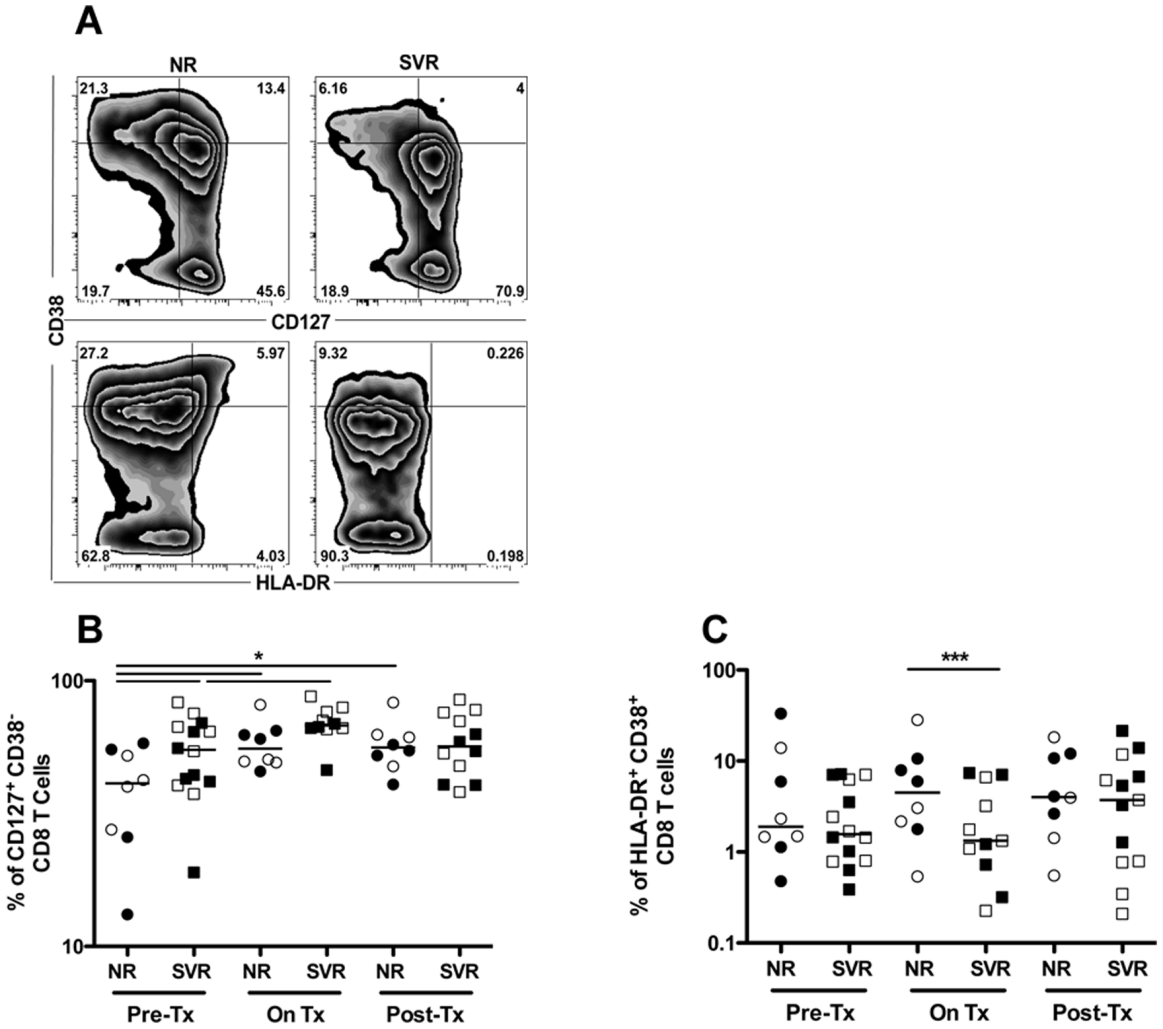
Sustained virologic response to IFN-α therapy correlates with higher baseline and on treatment expression of CD127 while non-response correlates with increased CD8 T cell activation. Resting and activated CD8 T cells were defined as CD127^+^CD38^neg^ and HLA-DR^+^CD38^+^ CD8^+^CD3^+^ lymphocytes, respectively, and monitored longitudinally in NR (n = 8) and SVR (n = 14) HCV/HIV co-infected patients before, during and after IFN-α therapy. (A) Representative flow cytometry plots for one patient from each group NR or SVR at baseline. (B) Longitudinal expression of CD127 on CD38^neg^ CD8 T cells. (C) Longitudinal activation of CD8 T cells measured as percent of HLA-DR^+^CD38^+^ CD8 T cells. P-values were calculated using a two-tailed Mann Whitney U test to compare NR to SVR patients. Wilcoxon signed rank test was used to perform longitudinal statistical analysis. Open symbols represent patients of group A and closed symbols represent patients of group B. (* p<0.05, *** p<0.001).

As described above, we observed a reduction in total CD8 T cell counts in the SVR group during treatment. Therefore, we proceeded to investigate whether this was accompanied by a change in the distribution of resting and activated CD8 T cells identified as CD127^+^CD38^neg^ and HLA-DR^+^CD38^+^, respectively [39] (Figure 2A). We investigated if the outcome of HCV therapy can be predicted by the frequency of CD127^+^CD38^neg^ CD8 T cells. The frequency of resting CD8 T cells detected in SVR patients was significantly higher than in NR patients at baseline (p = 0.03) (Figure 2B). This cell subset significantly increased in frequency in SVRs during therapy (p = 0.03) then declined back to baseline levels afterwards. In NRs, although the frequency of CD127^+^CD38^neg^ CD8 T cells was lower than in SVRs at baseline, it still increased during therapy and remained elevated afterwards (p = 0.02 and p = 0.04, respectively). This may reflect preferential differentiation and/or selection of this cell subset or their resistance to IFN-α induced apoptosis.

Next we evaluated the effect of IFN-α therapy on the activation status of CD8 T cells by evaluating co-expression of HLA-DR and CD38 *ex-vivo* as was previously described during HIV mono-infection [40]. There was no significant difference in the frequency of activated CD8 T cells between NR and SVR patients at baseline (Figure 2C). However, the frequency of this cell subset increased in the NR patients during therapy and was significantly higher than in the SVR patients (p = 0.0003). Although it remained relatively high after therapy, there was no significant difference between the NR and SVR group.

### Non-response to IFN-α therapy correlates with increased CD8 T cell exhaustion at baseline

**Figure 3:**
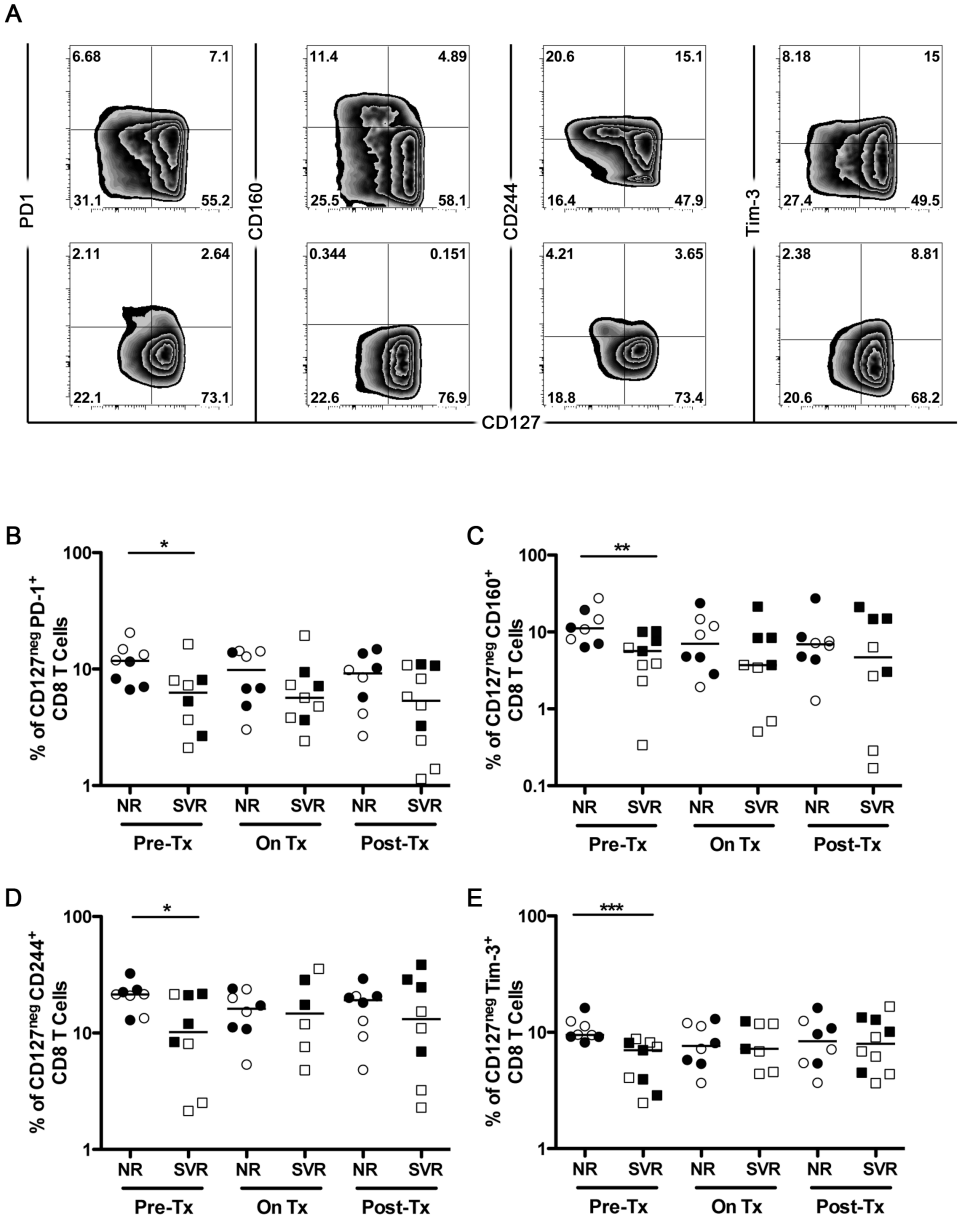
Non response to IFN-α therapy correlates with increased CD8 T cell exhaustion at baseline. Expression of the indicated activation/exhaustion molecules on CD8 T cells was monitored longitudinally in NR (n = 8) and SVR (n = 10) HCV/HIV co-infected patients before, during and after HCV therapy. (A) Representative flow cytometry plots for one patient from each group NR (top panel) and SVR (bottom panel) demonstrating expression of the different markers on total CD8 T cells (gated on viable CD8^+^CD3^+^ lymphocytes). (B-E) CD8 activation/exhaustion was measured by the absence of CD127 and the expression of the activation/exhaustion markers HLA-DR, PD-1, CD160, CD244 and Tim-3. P-values were calculated using a two-tailed Mann Whitney U test. Open symbols represent patients of group A and closed symbols represent patients of group B. (* p<0.05, ** p<0.01, *** p<0.001).

Similar to our analysis of CD4 T cells, we sought to determine whether IFN-α treatment induces exhaustion of CD8 T cells. Since CD127 is considered a marker of functional memory T cells [33], [41], we examined its expression in relation to the T cell inhibitory receptors PD1, CD160, CD244 and Tim-3 (Figure 3A). Cells expressing both CD127 and inhibitory receptors could represent heterogeneous population of recently activated effector memory T cells and so they were excluded from analysis. Low levels of CD127 expression were coupled with elevated expression of exhaustion markers on CD8 T cells *ex-vivo* (Figure 3A). The frequency of CD8 T cells expressing these inhibitory receptors was significantly elevated in NR patients as compared to SVRs at baseline, suggesting a higher level of exhaustion. NRs exhibited nearly 2 fold more CD127^neg^PD1^+^, CD127^neg^CD160^+^, CD127^neg^CD244^+^ and CD127^neg^Tim-3^+^ CD8 T cells than SVR patients (p = 0.03, p = 0.01, p = 0.05 and p = 0.01, respectively) (Figure 3B-E). As CD127 expression increased in response to IFN-α therapy (Figure 2B), we observed a decline in the expression of these inhibitory receptors in NR patients. We thus concluded that the response to IFN-α therapy correlates with the exhaustion status of CD8 T cells at baseline and that IFN-α may trigger death or relocalization of these exhausted/activated T cells into the liver.

### Sustained virologic response to IFN-α therapy correlates with higher HIV- and HCV-specific proliferation of CD4 and CD8 T cells at baseline

**Figure 4:**
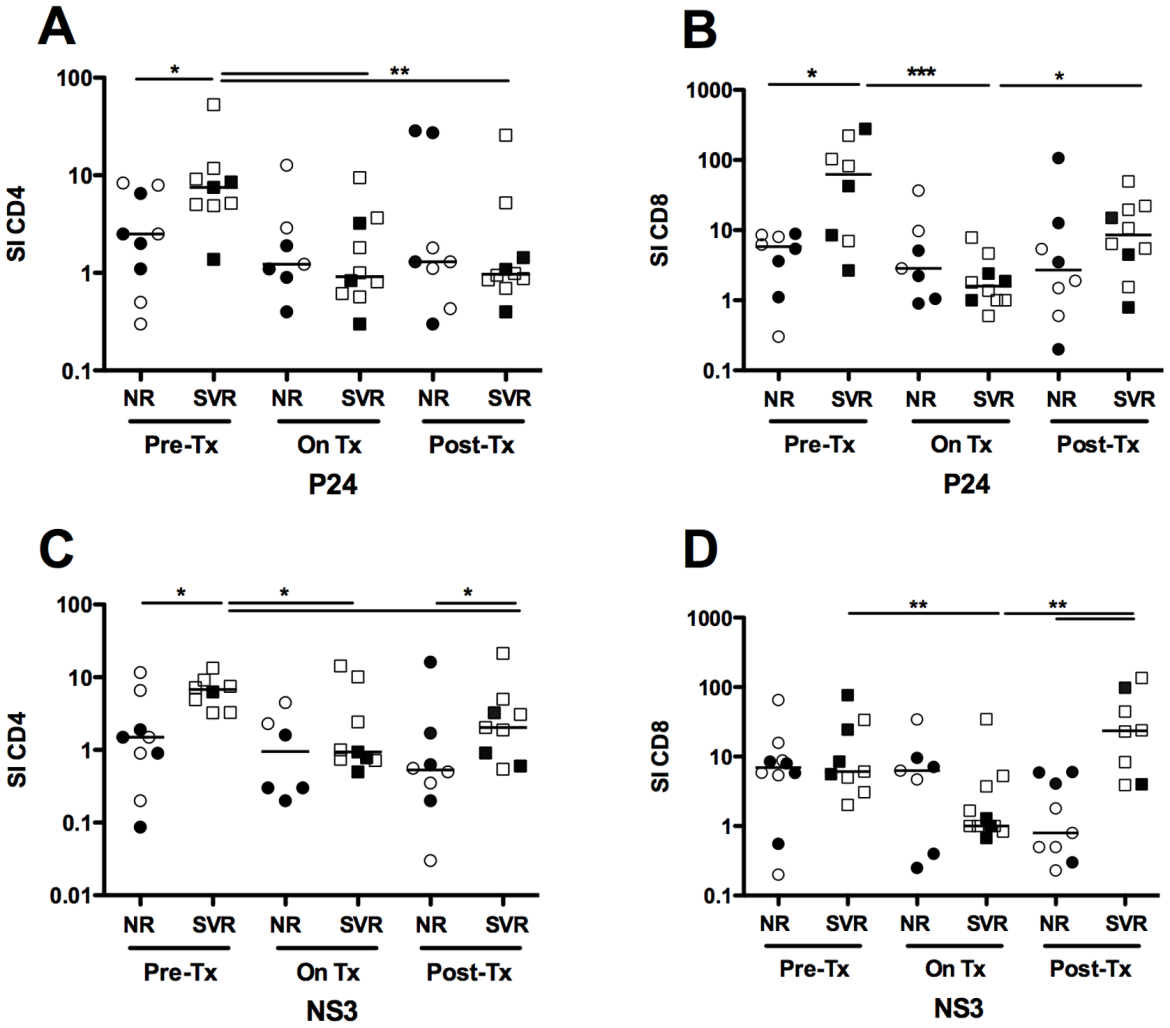
Sustained virologic response to IFN-α therapy correlates with higher HIV- and HCV-specific proliferation of CD4 and CD8 T cells at baseline. Proliferation of HIV- and HCV-specific CD4 and CD8 T cells in response to HIV P24 (A, B) and HCV NS3 (C, D) antigens was measured in NR (n = 9) and SVR (n = 11) HCV/HIV co-infected patients before, during and after IFN-α therapy. Briefly, patient PBMCs were labelled with CFSE and stimulated with 1 ug/ml of the indicated antigens for 6 days then stained as described in Materials and Methods. Proliferating antigen-specific T cells were identified by gating on viable CFSE^low^CD4^+^CD3^+^ (A, C) or CFSE^low^CD8^+^CD3^+^ lymphocytes (B, D). Stimulation Index (SI) was calculated using the following formula: % CFSE^low^ (antigen stimulated)/% CFSE^low^ (unstimulated). P-values were calculated using a two-tailed Mann Whitney U test to compare NR to SVR patients. Wilcoxon signed rank test was used to perform longitudinal statistical analysis. Open symbols represent patients of group A and closed symbols represent patients of group B. (* p<0.05, ** p<0.01, *** p<0.001).

Next, we sought to examine how IFN-α therapy influences HCV- and HIV-specific T cell responses. First we used MHC class I tetramers to monitor the frequency of HCV-, HIV- and CMV-specific T cells. No HCV-specific CD8 T cells directed against common HCV tetramers targeting immune-dominant HCV-epitopes within the core, NS3 and NS5b regions were detected at baseline. This is consistent with reports in the literature demonstrating that HCV-specific T cells are barely detectable in the peripheral blood during chronic infection as they may be of low frequency or localized to the liver [42] Although, HIV-specific T cells were detectable at baseline, they became undetectable during IFN-α therapy (data not shown). Co-expression of the exhaustion markers PD1 and Tim-3 (p = 0.03) as well as expression of CD160 (p = 0.02) were higher on HIV-specific than on CMV-specific CD8 T cells (Figure S4B). Analysis of inhibitory receptor expression on HIV-specific CD8 T cells at baseline demonstrated thus an advanced exhaustion status [43], which may explain their disappearance from the periphery with the initiation of therapy (Figure S4A). We thus proceeded with an alternate strategy to examine the proliferative capacity of antigen-specific T cells. The proliferation of CD4 and CD8 T cells was assessed *in vitro* using a CFSE-dilution based assay following stimulation with HCV (NS3) and HIV (p24) recombinant viral proteins as described in Materials and Methods. The baseline proliferation of antigen-specific T cells was generally higher in SVR than in NR patients for CD4 (p = 0.04 and p = 0.02 for p24 and NS3, respectively) (Figure 4A and 4C) and CD8 T cells (p = 0.02 for p24) (Figure 4B). IFN-α treatment induced a reduction in the proliferative capacity of the limited number of HCV- and HIV-specific CD8 T cells in NR patients during therapy and did not fully recover afterwards (p = 0.05) (Figure 4B, 4C). SVR patients exhibited reduced proliferation of HCV- and HIV-specific CD4 T cells (p = 0.01 and p = 0.01 for p24 during and after therapy, respectively and p = 0.02 and p = 0.01 for NS3 during and after therapy, respectively). The proliferative capacity of CD8 T cells was also reduced during therapy in SVR patients (p = 0.01 and p = 0.01 for p24 and NS3, respectively). After therapy, the antigen specific CD4 and CD8 T cells recovered their proliferative capacity although not completely to baseline levels (p = 0.04 and p = 0.01 for p24 and NS3, respectively). Moreover, proliferation of NS3 and p24 specific CD4 T cells correlated positively with the frequency of CD127 CD4 T cells prior to IFN-α therapy (Figure S5). In summary, SVR patients were characterized by higher proliferation at baseline and although IFN-α therapy induced a reduction in the proliferative capacity of CD4 T cells and CD8 T cells, this function was partially restored after the termination of therapy. In contrast, proliferation of CD4 and CD8 T cells in NR patients was low at baseline and after therapy, decreased during treatment and recovered less after termination of therapy.

## Discussion

We demonstrated that CD127 expression was coupled with lower T cell exhaustion status and fibrosis. Moreover, a higher virus-specific proliferative capacity correlated with responsiveness to IFN-α therapy. The cooperation between adaptive immune T cells and IFN-α to control HCV replication may explain ability of patients with strong expression of CD127 to a successful HCV therapy. In contrast, non-response to IFN-α was associated with T cell exhaustion, defined here by the expression of inhibitory receptors such as PD-1, CD160, CD244 or Tim-3 [43] and reduced expression of CD127 [44] at baseline.

IFN-α therapy induced a slight decrease in the frequency of CD127^+^CD4 T cells, an increase in activation and a reduction in proliferation of HIV- and HCV-specific T cells in SVR patients. These effects then recovered slightly but not to baseline levels at 6 months following end of treatment. The slight decline in CD127 expression could be explained by persistent activation of CD4 T cells in co-infected patients either directly due to residual viral replication despite being on cART or indirectly due to microbial translocation. CD127 expression on CD4 T cells also correlated inversely with liver fibrosis (despite reduction of APRI score in SVRs after IFN-α therapy). Tim-3 expression (or dual expression of PD1/Tim-3, data not shown) was less associated with liver fibrosis in this cohort than previously shown [45]. This may be due to the limited number of patients in our study, differences in the duration of cART or adherence to IFN-α therapy. Furthermore, It is also possible that functional CD127^high^ CD4 T cells were recruited to the liver and therefore may have reduced tissue damage by secretion of hepato-protective cytokines [46].

The reduction in CD4 T cell functions in SVR patients despite no significant change in their counts in the periphery could be due to a direct inhibitory effect of IFN-α [47], modulation of antigen presentation [48], reduced thymopoeisis [49] or an imbalance in the ratio between the inhibitory regulatory CD4 T cells and the inflammatory Th17 CD4 T cells as observed during pathogenic SIV infection [50] and primary HIV [51] or acute HCV infection [32]. Given that CD4 cell counts were already low at baseline, it is possible that they required longer time to fully recover fully their phenotype and functional levels.

As suggested by recent reports, IFN-α therapy can induce a number of HIV restriction factors and interferon stimulated genes (ISGs) that enhance clearance of the latent HIV proviral reservoir in CD4 T cells from co-infected patients [24], [52]. Induction of such genes may also disrupt CD4 T cell functions and impact their survival and thus contribute to limited recovery of these cells. Further investigations are required to determine if SVR is associated with a reduction in the cell associated HIV reservoir and functionality of HIV-specific CD4 T cells.

IFN-α therapy induced an overall reduction in the frequency of peripheral CD8 T cells only in SVR patients. This may be due to selective migration (or sequestration) of these activated T cells to the liver [53] and suggest that better T cell migration may be important for efficient antiviral responses. Further investigations examining the expression of chemokine receptors associated with localization to the liver such as CCR5, CXCR3 or CXCR6 [54]–[58] are needed. This may also be more relevant in the NR patients where CD8 T cells have a higher exhaustion status and may already be less responsive to IFN-α. Indeed, IFN-α hyper-responsiveness and increased expression of ISGs at baseline has been linked to dampened response to additional IFN-α stimulation upon therapy in HCV mono-infected individuals [59], [60].

We observed an increase in the frequency of CD127^+^ CD8 T cells in all treated patients. IFN has also been reported to increase expression of CD127 receptor on cell surface [61] and may indirectly favor its recycling [62], protein stabilization [63] or increase transcription of CD127 mRNA [64]–[67]. These observations suggest that IFN-α treatment does not affect the different T cell populations in the same way. Moreover, high expression of anti-apoptotic molecules such as Bcl-2 [33] may protect CD127^+^ T cells from side effects associated with IFN-α/RBV-based therapy.

The HCV- and HIV-specific proliferative T cell response was low in NR patients at baseline. This correlates with the general increase in the exhaustion status of CD4 and CD8 T cells in NR co-infected individuals. The fact that the response does recover partly after the end of therapy is promising as it suggests that IFN-α therapy has less deleterious effects on HIV-specific responses and that ART intensification using IFN-α could be used to eliminate the latent HIV reservoir and achieve better cure of HIV infection [25] in co-infected individuals without risk of developing severe immunodeficiency and/or opportunistic infections.

Although we attempted to differentiate patients based on the order of infections i.e. HIV first versus HCV first, we did not observe any remarkable difference between the two groups. Our data suggest that once chronic co-infection with both viruses is established, there is very little difference as to what the order of infection was and other immune factors like CD4 T cell counts and immune activation could be the key determinant of response to therapy.

IFN-α will remain a major component of HCV therapy in combination with newly developed DAAs that have demonstrated better response rates in co-infected patients [3], [68]. The use of adjuvants such as IL-7 [69] that may counteract some of the lymphopenic effects of IFN-α may sustain functionality of CD4 and CD8 T cells and improve treatment efficacy. We have shown in this study that baseline levels of CD127 expression and antigen specific proliferation may together provide good predictors of the response to therapy. The role of IL28B single nucleotide polymorphism (SNP), an established predictor of IFN-α therapy outcome [70], could not be addressed in this study due to the limited number of patients. A more expanded study examining the influence of IL28B SNP on HIV- and HCV-specific T cell immunity in the co-infected population and elimination of the latent HIV proviral reservoir is warranted.

## Supporting Information

Figure S1 **IFN-α therapy induces a reduction in CD8 T cell counts in SVR patients.** Total lymphocytes and CD4 and CD8 T cell counts were measured as part of the clinical follow-up of patients at baseline, during and 6 months after the termination of IFN-α therapy in NR (n = 9) and SVR (n = 17) HCV/HIV co-infected patients. Wilcoxon signed rank test was used to perform statistical analysis. P-values were calculated using a two-tailed Mann Whitney U test to compare NR with SVR patients but no statistical differences were observed. (* p<0.05)
(TIF)

Figure S2 **IFN-α therapy induces a reduction in APRI score in SVR patients.** APRI score was measured as part of the clinical follow-up of patients at baseline, during and 6 months after the termination of IFN-α therapy in NR (n = 9) and SVR (n = 19) HCV/HIV co-infected patients. Wilcoxon signed rank test was used to perform statistical analysis. Open symbols represent patients of group A and closed symbols represent patients of group B.
(TIF)

Figure S3 **Liver fibrosis correlates with baseline expression of CD127 and Tim-3 on CD4 T cells.** Expression of CD127 (A-C) and Tim-3 (B-D) on total CD4 T cells is associated with clinical parameter of liver injury in HCV/HIV co-infected patients before (A-B) or after IFN-α therapy (C). Correlations between memory (A-C) or exhaustion (B-D) markers on CD4 T cells and their corresponding APRI score were calculated using the Pearson correlation test. NR and SVR patients are represented respectively by closed circles (n = 6) and squares (n = 12).
(TIF)

Figure S4 **Baseline exhaustion status of HIV–specific CD8 T cells.** The frequency and phenotype of CMV and HIV-specific CD8 T cells was measured using the following MHC class I CMV (A2/pp65 and B7/pp65) and HIV (A2/p17, A2/p24, A2/Nef, B7/p24 and B7/Nef)–specific tetramers. (A) Representative flow cytometry data demonstrating detailed phenotypic characterization of CMV- and HIV-specific CD8 T cells using tetramers at baseline. Cells were gated on tetramer^+^CD8^+^CD3^+^ viable lymphocytes (black dot plot) overlaid on total CD8^+^CD3^+^ viable lymphocytes (grey contour plot). (B) Advanced exhaustion status of HIV-specific CD8 T cells as compared to CMV in co-infected patients at baseline irrespective of treatment outcome. Expression of the inhibitory receptors PD1, Tim-3 and CD160 was assessed on the surface of CMV- and HIV-specific CD8 T cells as identified by tetramers in panel A (n = 16 and n = 39, respectively). P-values were calculated using a two-tailed Mann Whitney U test. (* p<0.05, ** p<0.01).
(TIF)

Figure S5 **Virus-specific CD4 T cell proliferation correlates with baseline expression of CD127 on CD4 T cells.** HIV-specific (A-C) and HCV-specific (B-D) CD4 T cell proliferation correlates with expression of CD127 on total CD4 T cells in HCV/HIV co-infected patients before (A-B) but not after IFN-α therapy (C-D). Correlations were tested using the Pearson correlation test. NR and SVR patients are represented by closed circles (n = 3) and squares (n = 10), respectively.
(TIF)
