# Supplementary figures and images for: CD127 Expression, Exhaustion Status and Antigen Specific Proliferation Predict Sustained Virologic Response to IFN in HCV/HIV Co-Infected Individuals
Source: PLoS One. 2014 Jul 9;9(7):e101441. doi: 10.1371/journal.pone.0101441 (PMC4090061; doi:10.1371/journal.pone.0101441)

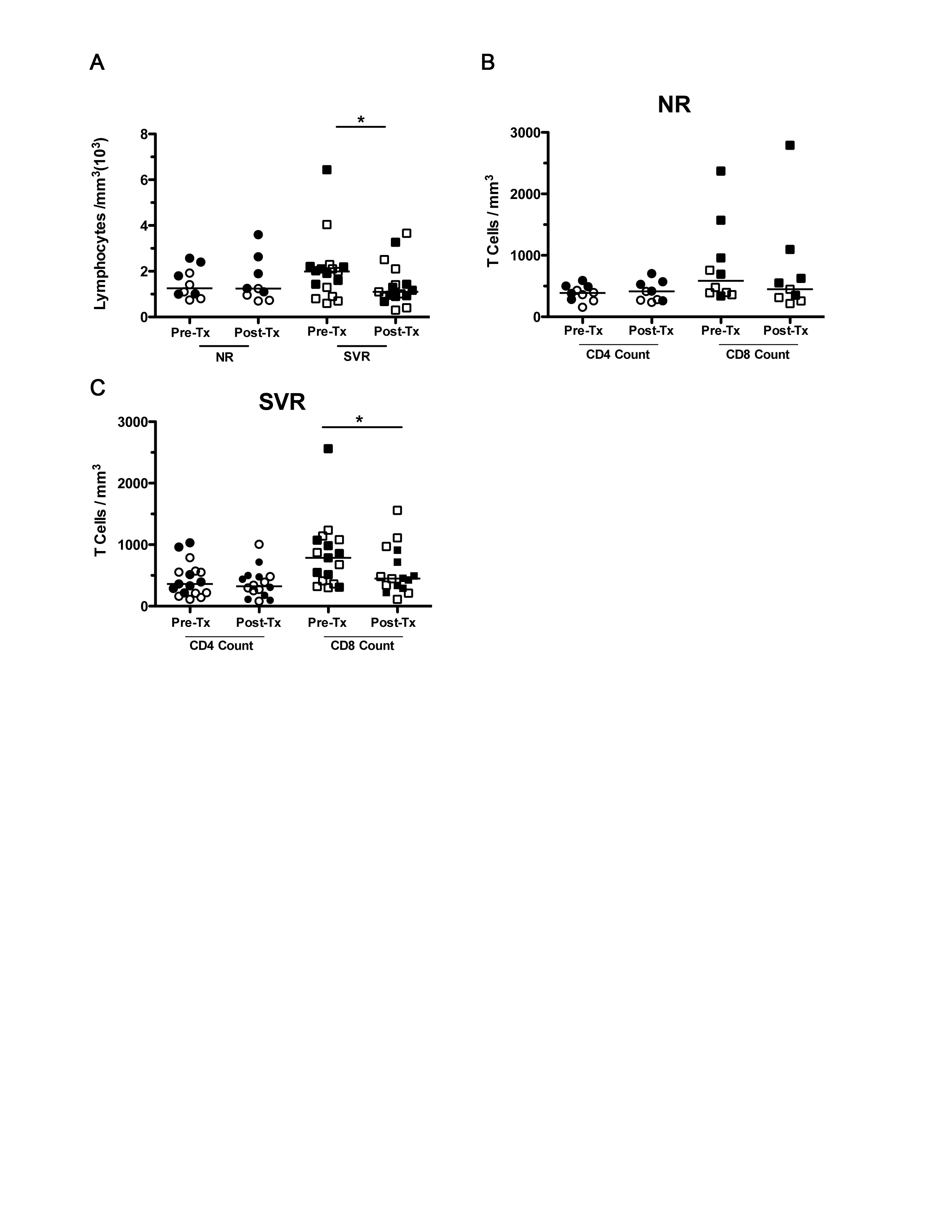

Supplement: Figure S1 — IFN-α therapy induces a reduction in CD8 T cell counts in SVR patients. Total lymphocytes and CD4 and CD8 T cell counts were measured as part of the clinical follow-up of patients at baseline, during and 6 months after the termination of IFN-α therapy in NR (n = 9) and SVR (n = 17) HCV/HIV co-infected patients. Wilcoxon signed rank test was used to perform statistical analysis. P-values were calculated using a two-tailed Mann Whitney U test to compare NR with SVR patients but no statistical differences were observed. (* p<0.05) (TIF) [file pone.0101441.s001.tif]

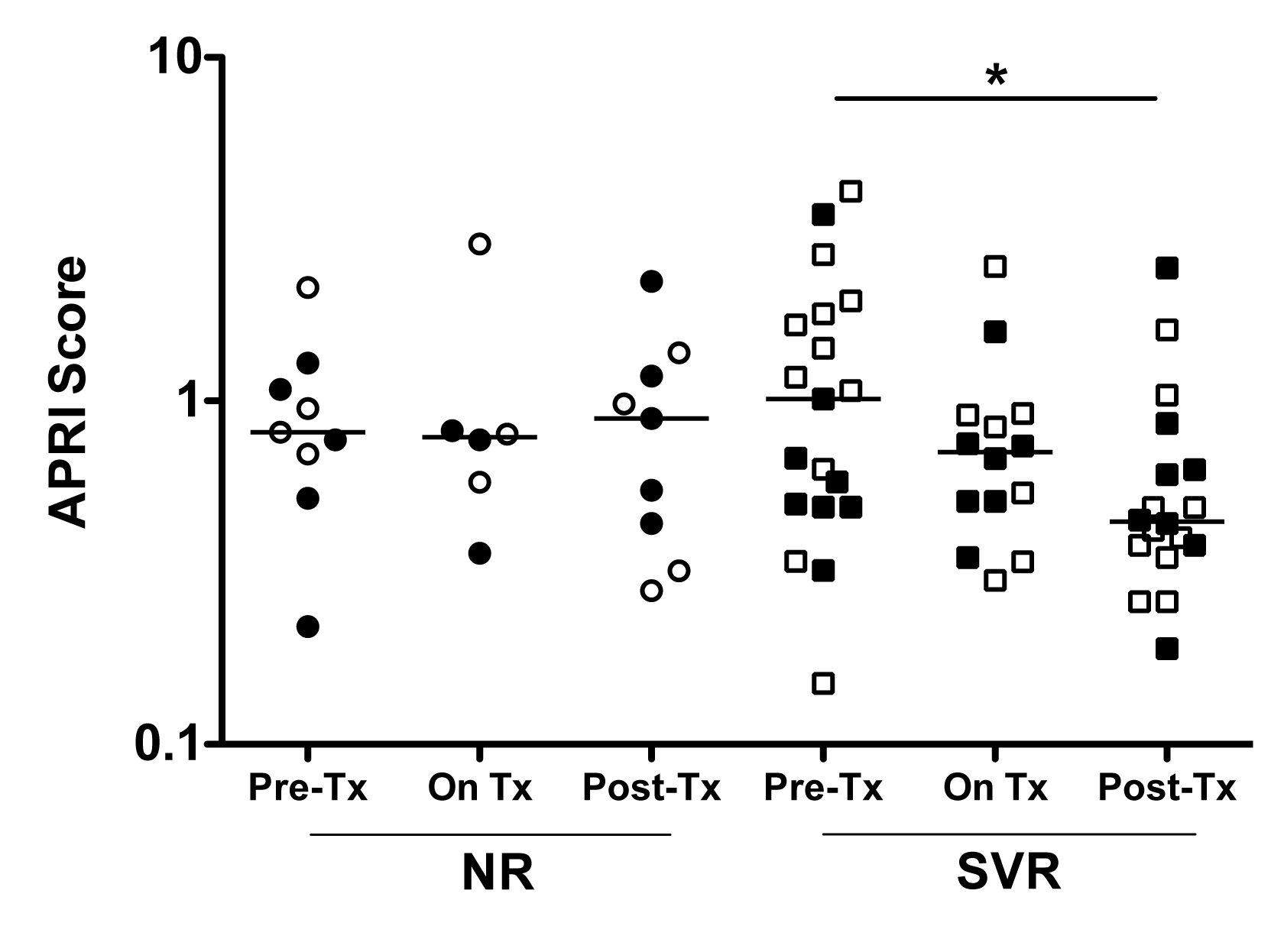

Supplement: Figure S2 — IFN-α therapy induces a reduction in APRI score in SVR patients. APRI score was measured as part of the clinical follow-up of patients at baseline, during and 6 months after the termination of IFN-α therapy in NR (n = 9) and SVR (n = 19) HCV/HIV co-infected patients. Wilcoxon signed rank test was used to perform statistical analysis. Open symbols represent patients of group A and closed symbols represent patients of group B. (TIF) [file pone.0101441.s002.tif]

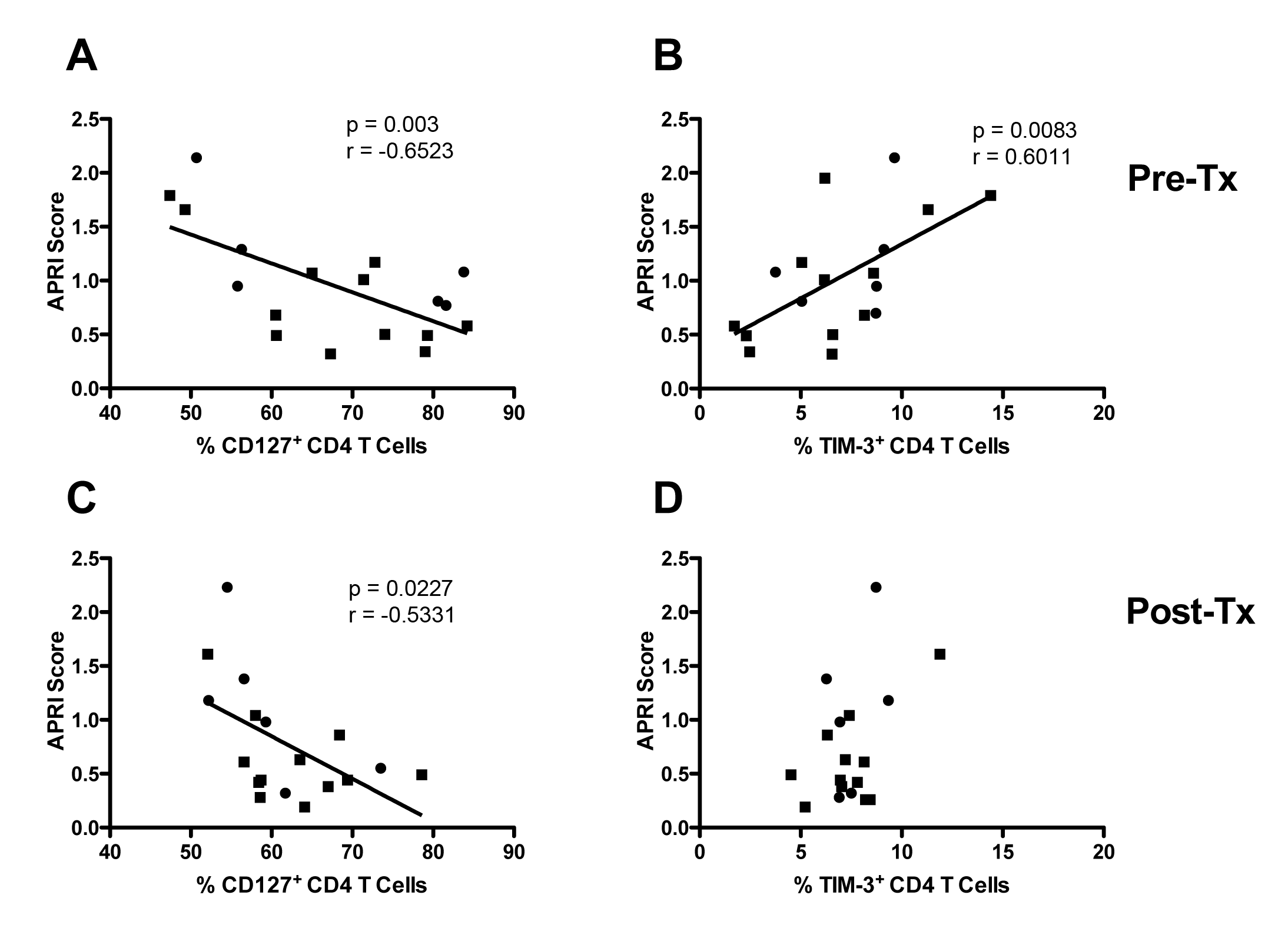

Supplement: Figure S3 — Liver fibrosis correlates with baseline expression of CD127 and Tim-3 on CD4 T cells. Expression of CD127 (A-C) and Tim-3 (B-D) on total CD4 T cells is associated with clinical parameter of liver injury in HCV/HIV co-infected patients before (A-B) or after IFN-α therapy (C). Correlations between memory (A-C) or exhaustion (B-D) markers on CD4 T cells and their corresponding APRI score were calculated using the Pearson correlation test. NR and SVR patients are represented respectively by closed circles (n = 6) and squares (n = 12). (TIF) [file pone.0101441.s003.tif]

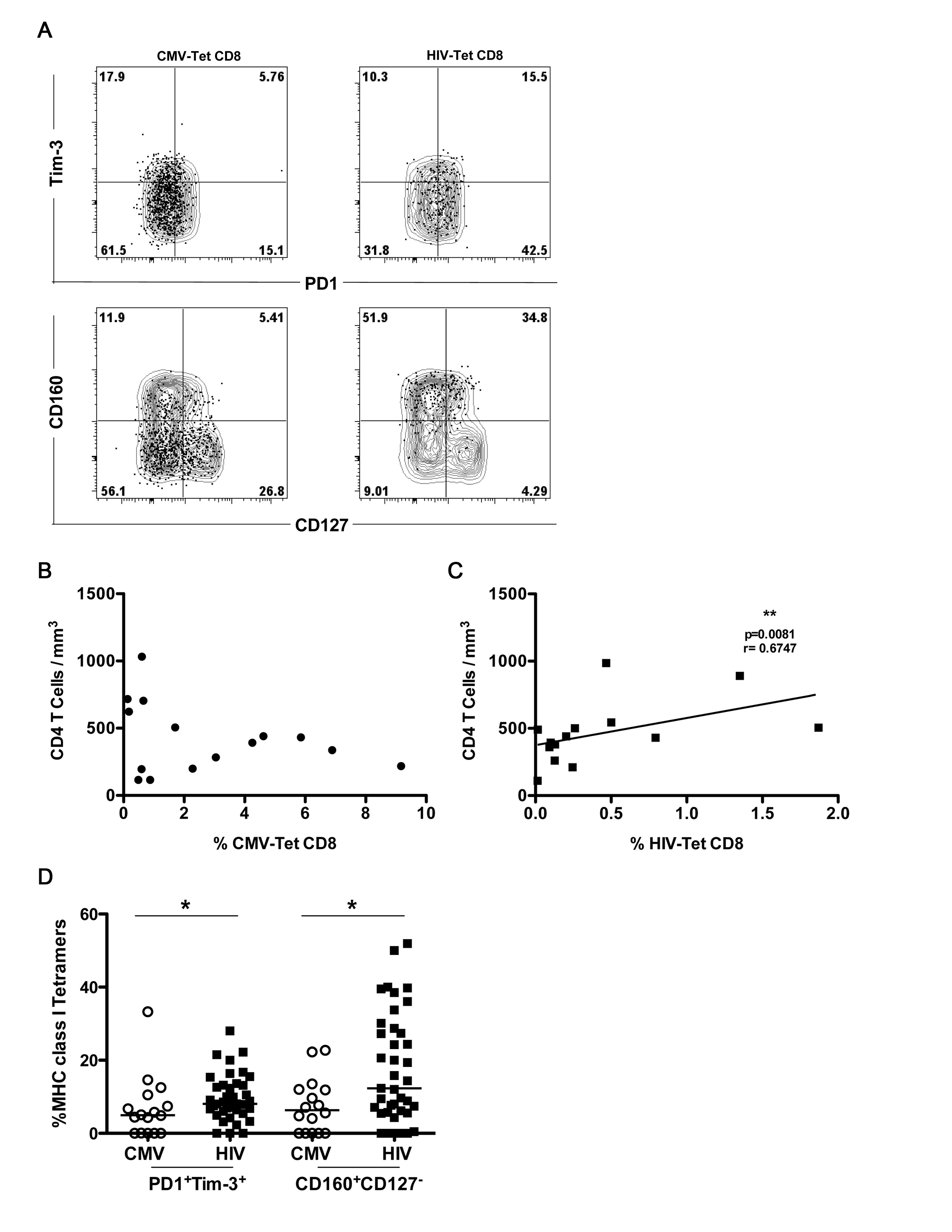

Supplement: Figure S4 — Baseline exhaustion status of HIV–specific CD8 T cells. The frequency and phenotype of CMV and HIV-specific CD8 T cells was measured using the following MHC class I CMV (A2/pp65 and B7/pp65) and HIV (A2/p17, A2/p24, A2/Nef, B7/p24 and B7/Nef)–specific tetramers. (A) Representative flow cytometry data demonstrating detailed phenotypic characterization of CMV- and HIV-specific CD8 T cells using tetramers at baseline. Cells were gated on tetramer+CD8+CD3+ viable lymphocytes (black dot plot) overlaid on total CD8+CD3+ viable lymphocytes (grey contour plot). (B) Advanced exhaustion status of HIV-specific CD8 T cells as compared to CMV in co-infected patients at baseline irrespective of treatment outcome. Expression of the inhibitory receptors PD1, Tim-3 and CD160 was assessed on the surface of CMV- and HIV-specific CD8 T cells as identified by tetramers in panel A (n = 16 and n = 39, respectively). P-values were calculated using a two-tailed Mann Whitney U test. (* p<0.05, ** p<0.01). (TIF) [file pone.0101441.s004.tif]

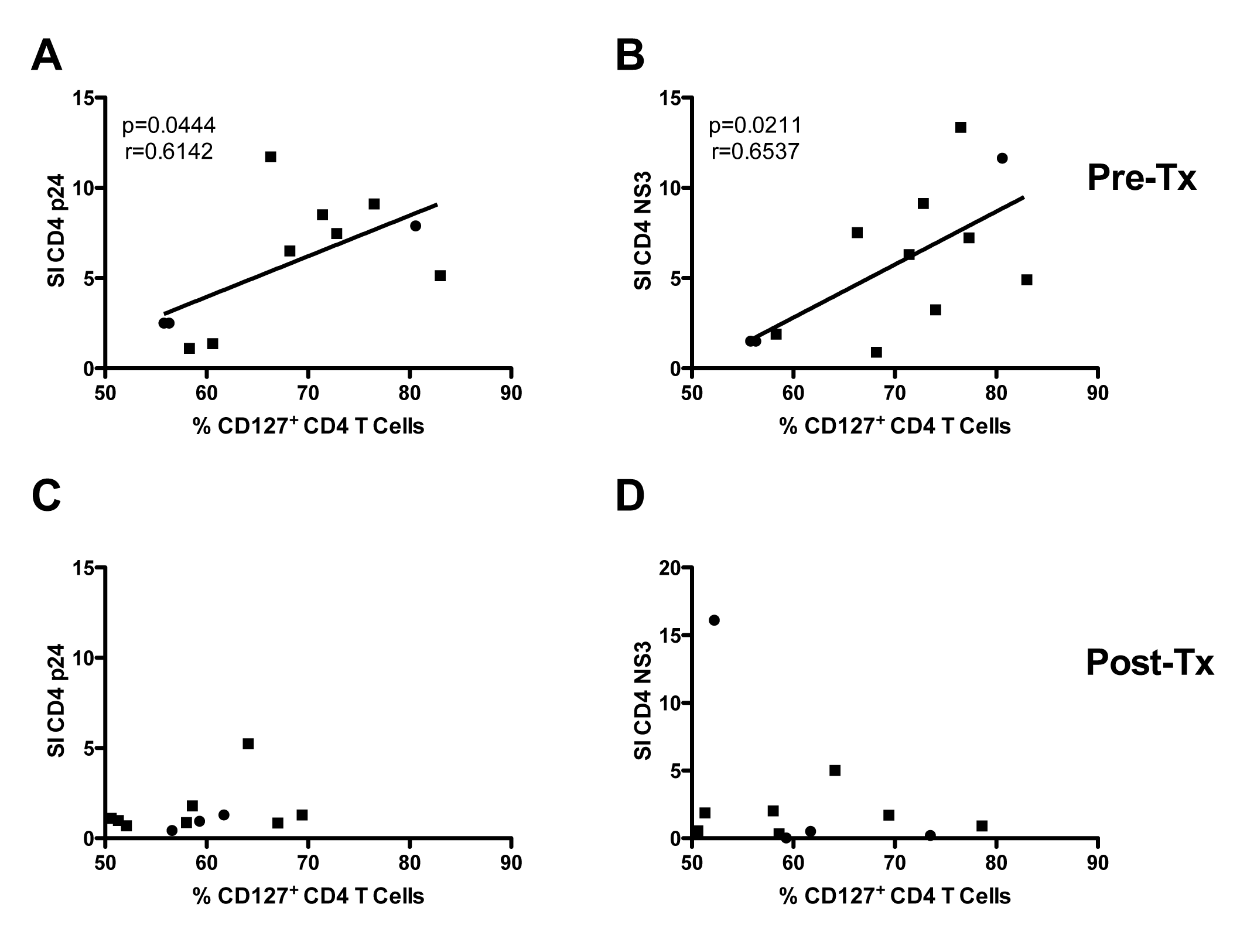

Supplement: Figure S5 — Virus-specific CD4 T cell proliferation correlates with baseline expression of CD127 on CD4 T cells. HIV-specific (A-C) and HCV-specific (B-D) CD4 T cell proliferation correlates with expression of CD127 on total CD4 T cells in HCV/HIV co-infected patients before (A-B) but not after IFN-α therapy (C-D). Correlations were tested using the Pearson correlation test. NR and SVR patients are represented by closed circles (n = 3) and squares (n = 10), respectively. (TIF) [file pone.0101441.s005.tif]
